# Quantification of *in vivo* transverse relaxation of glutamate in the frontal cortex of human brain by radio frequency pulse-driven longitudinal steady state

**DOI:** 10.1371/journal.pone.0215210

**Published:** 2019-04-17

**Authors:** Ningzhi Li, Linqing Li, Yan Zhang, Maria Ferraris Araneta, Christopher Johnson, Jun Shen

**Affiliations:** 1 Section on Magnetic Spectroscopy, National Institute of Mental Health, National Institutes of Health, Bethesda, Maryland, United States of America; 2 Functional Magnetic Resonance Imaging Core Facility, National Institute of Mental Health, National Institutes of Health, Bethesda, Maryland, United States of America; 3 Magnetic Resonance Spectroscopy Core, National Institute of Mental Health, National Institutes of Health, Bethesda, Maryland, United States of America; INSERM, FRANCE

## Abstract

**Purpose:**

The principal excitatory neurotransmitter glutamate plays an important role in many central nervous system disorders. Because glutamate resides predominantly in glutamatergic neurons, its relaxation properties reflect the intracellular environment of glutamatergic neurons. This study developed an improved echo time-independent technique for measuring transverse relaxation time and demonstrated that this radio frequency (RF)-driven longitudinal steady state technique can reliably measure glutamate transverse relaxation in the frontal cortex, where structural and functional abnormalities have been associated with psychiatric symptoms.

**Method:**

Bloch and Monte Carlo simulations were performed to improve and optimize the RF-driven, longitudinal, steady-state (MARzss) technique to significantly shorten scan time and increase measurement precision. Optimized four-flip angle measurements at 0°,12°, 24°, and 36° with matched repetition time were used in nine human subjects (6F, 3M; 27–49 years old) at 7 Tesla. Longitudinal and transverse relaxation rates for glutamate were measured from a 2 x 2 x 2 cm^3^ voxel placed in three different brain regions: gray matter-dominated medial prefrontal lobe, white matter-dominated left frontal lobe, and gray matter-dominated occipital lobe.

**Results:**

Compared to the original MARzss technique, the scan time per voxel for measuring glutamate transverse relaxation was shortened by more than 50%. In the medial frontal, left frontal, and occipital voxels, the glutamate T_2_ was found to be 117.5±12.9 ms (mean ± standard deviation, n = 9), 107.3±12.1 (n = 9), and 124.4±16.6 ms (n = 8), respectively.

**Conclusions:**

The improvements described in this study make the MAR_ZSS_ technique a viable tool for reliably measuring glutamate relaxation from human subjects in a typical clinical setting. It is expected that this improved technique can be applied to characterize the intracellular environment of glutamatergic neurons in a variety of brain disorders.

## Introduction

Glutamate (Glu) has several fundamentally important functions in the central nervous system (CNS). It is the principal excitatory neurotransmitter as well as a key metabolite linking carbon and nitrogen metabolism [1]. The dual roles of Glu as a neurotransmitter and as a by-product of the citric acid cycle are intricately connected. Because of Glu’s crucial role in the glutamatergic process and energy metabolism, in vivo magnetic resonance spectroscopy (MRS) has been widely used to study the function of Glu in basic neuroscience as well as in various brain disorders [2–7]. Indeed, altered Glu concentrations have been linked to many pathologies, including head trauma, pain, aging, and many neurodegenerative diseases [2–5]. In addition, abnormal glutamatergic activity has been associated with psychiatric disorders such as schizophrenia and depression, and the glutamatergic system is currently a key focus of developing novel treatments for those disorders [6, 7].

Because Glu resides predominantly in glutamatergic neurons rather than astroglial cells [1], its magnetic resonance relaxation properties reflect the intracellular environment of glutamatergic neurons. Thus, the ability to reliably measure Glu T_2_ relaxation using a clinical scanner may be useful for studying diseases characterized by abnormal glutamatergic activity. With regard to psychiatric disorders in particular, probing the intracellular environment of glutamatergic neurons in the frontal cortex—where structural lesions, disturbed function, and morphology are strongly associated with symptoms—would be expected to be quite useful in terms of disease characterization as well as providing potential glutamatergic targets for monitoring treatment. Furthermore, the ability to measure Glu relaxation times is important for quantifying Glu levels by in vivo MRS because many Glu MRS methods require medium-to-long echo times (TEs) [8,9].

Techniques for measuring the relaxation properties of the dominant singlets N-acetylaspartic acid (NAA), choline (Cho), and creatine (Cr) are relatively well established [10]. Marked changes in the transverse relaxation times of the singlets have been found in bipolar disorder and schizophrenia [11]. In contrast, very few studies have attempted to measure Glu relaxation times. Conventional techniques for transverse relaxation time measurements require varying TE, which often causes baseline variations, large signal reductions, and spectral overlapping partly due to scalar coupling modulations. Other TE-dependent effects—for instance, diffusion—also contribute to difficulties in measuring Glu T_2_ and the T_2_ of J-coupled spins in general.

We recently demonstrated that metabolite T_2_ can be measured without changing TE [12]. Instead of the conventional multi-TE approach, relaxation weighting (T_2_/T_1_) was generated using a radio frequency (RF)-driven longitudinal steady state technique in the newly proposed multiple flip angle pulse-driven ratio of longitudinal steady states (MARzss) method. Because T_1_ is measured separately, the metabolite T_2_ can be quantified without needing to change TE. Therefore, a single TE can be chosen for T_1_ and T_2_ measurement. In this context, an optimal TE can be chosen to detect a certain J-coupled metabolite (e.g., Glu) with minimal spectral interference from overlapping resonances with or without spectral editing.

As originally developed, the MARzss method was only used to measure Glu T_2_ from the occipital cortex of human brain. It used a train of interleaved RF and gradient pulses (iPFG) with fixed total duration and a long recycle delay (repetition time (TR) = 8.5 at 7 Tesla (7T)) [12].

In the present study, the duration of the iPFG train and TR were varied based on the flip angle (FA) of the RF pulses used for relaxation weighting preparation. Bloch and Monte Carlo simulations were used to determine an optimal FA and iPFG train-set for reliably measuring Glu T_2_, and significantly shorten total acquisition time. In the present study we made major improvements on the MARzss method and applied it to characterize Glu relaxation in the frontal cortex.

## Materials and methods

### Overview of the MARzss method

**Fig 1:**
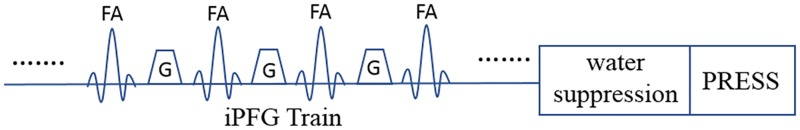
Diagram of the MARzss method. The first part is the interleaved RF and gradient pulses (iPFG) train, where FA is the flip angle of each radio frequency (RF) pulse and G is the field gradient inserted between two RF pulses in the train. G is applied in the z direction. Water suppression is applied following the iPFG train. Steady state longitudinal magnetization is measured by a point-resolved spectroscopy (PRESS) sequence optimized for Glu detection at 7 Tesla.

In the MARZss method, the steady state longitudinal magnetization weighted by T_1_/T_2_ is prepared by the iPFG train (Fig 1). A train of identical RF pulses is applied. Field gradients with the same amplitude and duration are inserted between RF pulses. Depending on the FA, each RF pulse in the iPFG train puts a portion of the longitudinal magnetization onto the transverse plane. The gradient immediately following the RF pulse dephases spin in the transverse plane before the next RF pulse is applied. After applying a considerable number of RF pulses and field gradients, the longitudinal magnetization is attenuated from its thermal equilibrium state (*M*_0_) to an RF-driven steady state [12]:

$$M_{zss}=M_{0}∙(1-\frac{AX}{\sqrt{1+A^{2}X^{2}}})$$ (1)

where $A=\sqrt{\frac{T_{1}}{T_{2}}}$ and $X=\text{tan}\left(\frac{FA}{2}\right)(0^{o}\leq FA\leq 90^{o})$. By measuring steady state magnetization (M_ZSS_) at several different FAs, a linear relationship between the relaxation weighting factor A of metabolites and the ratio of signal amplitudes (*R*_*zss*_) can be established:

$$R_{zss}=A∙X$$ (2)

where $R_{zss}=\frac{{{M_{0}-M}_{zss}}^{}}{\sqrt{M_{0}^{2}-{\left(M_{0}-M_{zss}\right)}^{2}}}$. The slope A of the above function can be solved by linear regression:

$$min||R_{zss}-A∙X{\left|\right|}^{2}$$ (3)

With the pre-determined metabolite T_1_ value, normally through TE-independent conventional techniques, such as inversion recovery, metabolite T_2_ values are determined:

$$T_{2}=T_{1}/A^{2}$$ (4)

Note that relaxation-weighting preparation and readout are uncoupled in the MARzss method. The RF-driven steady state magnetization M_ZSS_ can be measured without changing the readout TE and is independent of the readout sequence.

### Acquisition optimization

Bloch equation numerical simulations were performed to evaluate the spatial modulation and attenuation of magnetization in the longitudinal direction with different parameterizations. Linear regression of Eq [2] with R_ZSS_ measured at multiple FAs solves the T_1_/T_2_ ratio. Here, we quantified Glu T_2_ using four-FA measurements. The first FA was chosen to be zero (the smallest FA), and the last FA was chosen to have the furthest distance from 0 [13], while the signal intensity of Glu was not too small to be reliably determined. The signal measured at the last FA corresponded to the lowest signal to noise ratio (SNR). Therefore, T_2_ quantification accuracy was highly dependent on the intensity of the Glu signal with the largest FA.

Monte Carlo simulation was used to evaluate the estimation accuracy of Glu at different SNR levels. Metabolite concentrations, T_2_ values, and baseline values measured from a healthy volunteer (see below) were used to synthesize free induction decay (FIDs) using density matrix simulations. Random white noise with Gaussian distribution was added to the synthesized FIDs, and the noise level was determined from the in vivo data. This noise level was kept the same regardless of FA values. Quantification results from synthesized FIDs were computed using the LCModel fitting routine, developed in-house [14–16]. The whole procedure was repeated 100 times at 50 different SNR levels, corresponding to different attenuation ratios of Glu signal at 50 different FAs. The deviation from ground truth value at each SNR level was calculated. Because SNR increases when more signal averages are used, a contour plot as a function of FA and number of signal averages was generated. The numerical simulation of the spin density matrix was implemented in Java (Oracle Corporation, California) on an Eclipse Java Oxygen (www.eclipse.org)-integrated development environment (IDE). All other mathematical computations were programmed in MATLAB (R2016a).

### In vivo studies

Nine healthy volunteers (6F, 3M; ages 27–49 years) were recruited and scanned on a Siemens 7T scanner (Siemens Medical Solutions, Malvern, PA, USA) equipped with a 32-channel receiver head coil. All procedures were approved by the Institutional Review Board of the National Institute of Mental Health (NIMH). Written informed consent was obtained from each participant.

High resolution T_1_-weighted magnetization prepared rapid gradient echo (MPRAGE) images were acquired from each subject to position a 2×2×2 cm^3^ MRS voxel in three different locations: 1) a gray matter (GM)-dominated medial frontal lobe region; 2) a white matter (WM)-dominated left frontal lobe region; and 3) a GM-dominated medial occipital lobe region. Notably, the first and second locations have previously been linked to many psychiatric disorders [7,17,18]. Acquisition parameters were TR = 3 secs, TE = 3.9 ms, matrix = 256×256×256, resolution = 1x1x1 mm^3^. First- and second-order B_0_ field inhomogeneities within the selected voxel were corrected. Threshold-based segmentation was performed to calculate the percentage of GM, WM, and CSF in each voxel.

For in vivo measurement of Glu T_2_, TRs of 8.5 secs, 7.5 secs, 6.0 secs, and 4.5 secs were used for FA = 0, 12, 24, and 36 degrees, respectively. The corresponding iPFG train consisted of 750, 700, 550, and 350 identical sinc-Gauss RF pulses, respectively. The duration of each RF pulse was 4 ms and the inter-pulse delay was 10 ms. The interspersed gradient amplitude was 2 mT/m with a duration of 5 ms. A water suppression module, consisting of nine sinc-Gauss RF pulses with a duration of 9 ms and an inter-pulse delay of 12 ms [12] followed by a spoiling gradient after each RF pulse, was placed immediately after the iPFG train. The duration of all spoiling gradients was set to 1.2 ms. Relaxation-weighted spatially modulated longitudinal magnetization was acquired with a point-resolved spectroscopy (PRESS) sequence optimized for Glu detection at 7T [19]. Acquisition parameters of the PRESS sequence were TE_1_ = 69 ms, TE_2_ = 37 ms, data points = 2048, and number of averages = 16. To calibrate RF pulse angle, a simulated echo was used to select a thin bar across the center of the voxel and generate a one-dimensional image of this bar. The angle of the first pulse (which was nonselective) was varied until a 180 null was generated at the center of the voxel [20]. Unsuppressed water signal was also acquired for each FA. The total scan time for measuring T_1_/T_2_ using four FA with 16 averages was ~7 mins per voxel. Glu T_1_ was determined using the conventional inversion recovery method. Inversion recovery times of 255, 355, 455, 655, 1225, 1825, 2825, 3825, and 5025 ms were used.

Metabolite concentrations were determined using an LCModel fitting routine developed in-house [14–16]. To generate the basis set for LCModel fitting, the exact PRESS readout sequence used in the in vivo studies was simulated using the highly accelerated one-dimensional projection technique [21]. Effects of scalar evolution of the Glu signal during iPFG train were quantified and results are included in the supporting information. Density matrix simulated metabolites used in the LCModel included NAA, N-acetylaspartylglutamate (NAAG), total creatine (tCr), total choline (tCho), aspartate (ASP), Glu, glutamine (Gln), glutathione (GSH), γ-aminobutyric acid (GABA), and myo-inositol (mI). For each FA, the 32-channel water-suppressed FIDs were averaged from 16 acquisitions and subsequently combined into single-channel FIDs using a generalized least squares method [22]. The single unsuppressed water FID was similarly processed and used to correct eddy current effects. All spectra data were zero filled eight times and apodized using a combination of 3Hz Lorentzian and 3Hz Gaussian functions before Fourier transform. The Lorentzian and Gaussian Zero-order phase, first-order phase, and spline baselines were coded as fitting parameters in the fitting algorithm. Glu T_1_ values were pre-determined by fitting the inversion recovery data. Glu T_2_ values were subsequently determined by linearly fitting the in vivo signal amplitude ratio (Rzss, defined in Eq [2]) as a function of tan(FA/2). Delay due to water suppression was compensated as a slight magnetization recovery and corrected in Eq 1 [12]. Fitting coefficients of determination (R^2^) were calculated for each brain region of individual subjects.

## Results

**Fig 2:**
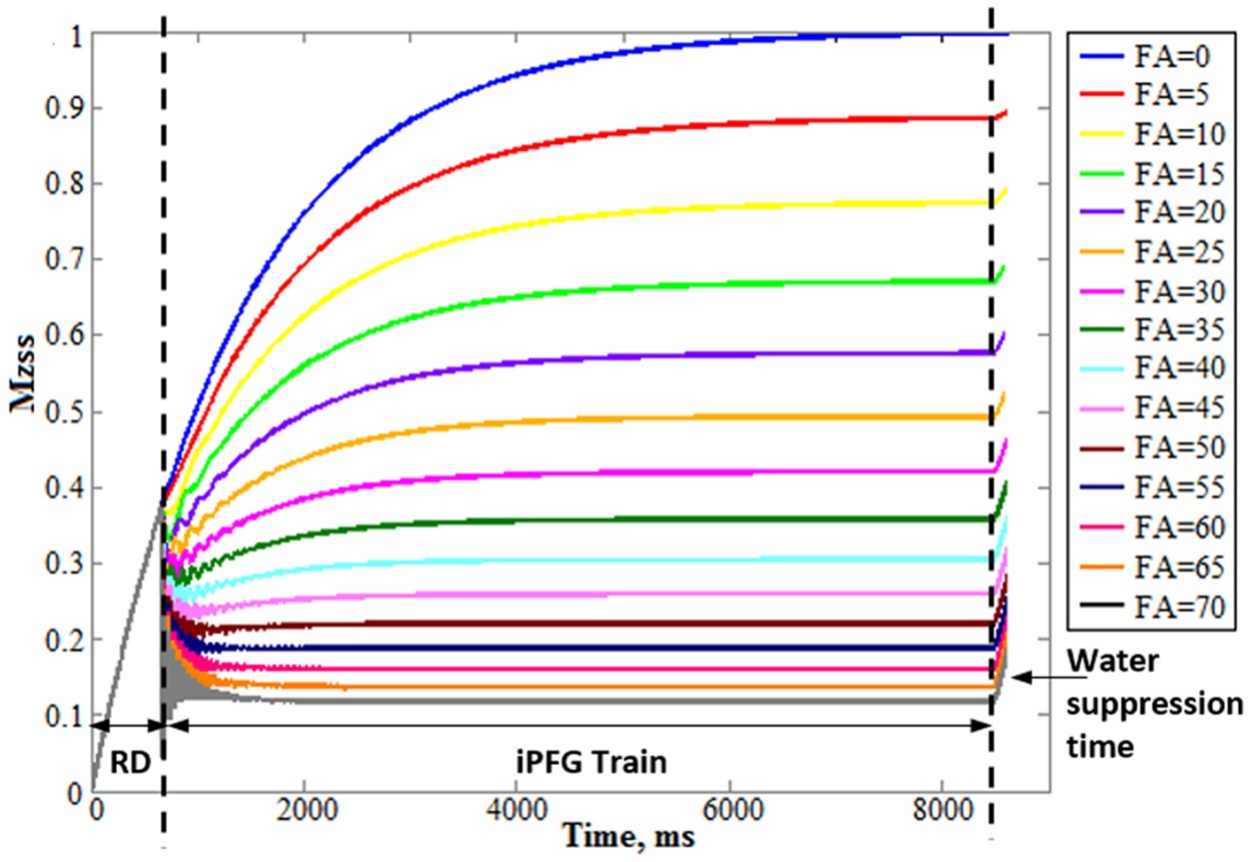
Bloch simulation of Mzss. Bloch simulation of longitudinal steady state magnetization (Mzss) temporal variation in one repetition time (TR) at different flip angle (FA) (unit: degree). Mzss achieved steady state much more quickly as FA increased. Short recovery of longitudinal magnetization during water suppression was compensated for in Eq 1. RD: recovery delay between PRESS and the interleaved RF and gradient pulses (iPFG) train.

Fig 2 displays the Bloch simulation of temporal variation of longitudinal magnetization at different FAs during one TR. As expected, the signal attenuated more from thermal equilibrium signal intensity as FA increased. The time needed for longitudinal magnetization to achieve steady state also varied for each FA. For example, only 4.5 secs of TR were required when the FA was at 36°. Overall, the time required for magnetization to achieve RF-driven steady state gradually decreased as FA increased. This indicates that a shorter TR can be used for a larger FA and, commensurately, that the total scan time can be significantly reduced by using different TR_min_ for different FAs; here, TR_min_ is defined as the minimum time required for magnetization to achieve RF-driven steady state (when the amplitude change of magnetization is less than 0.1%) in a single scan.

**Fig 3:**
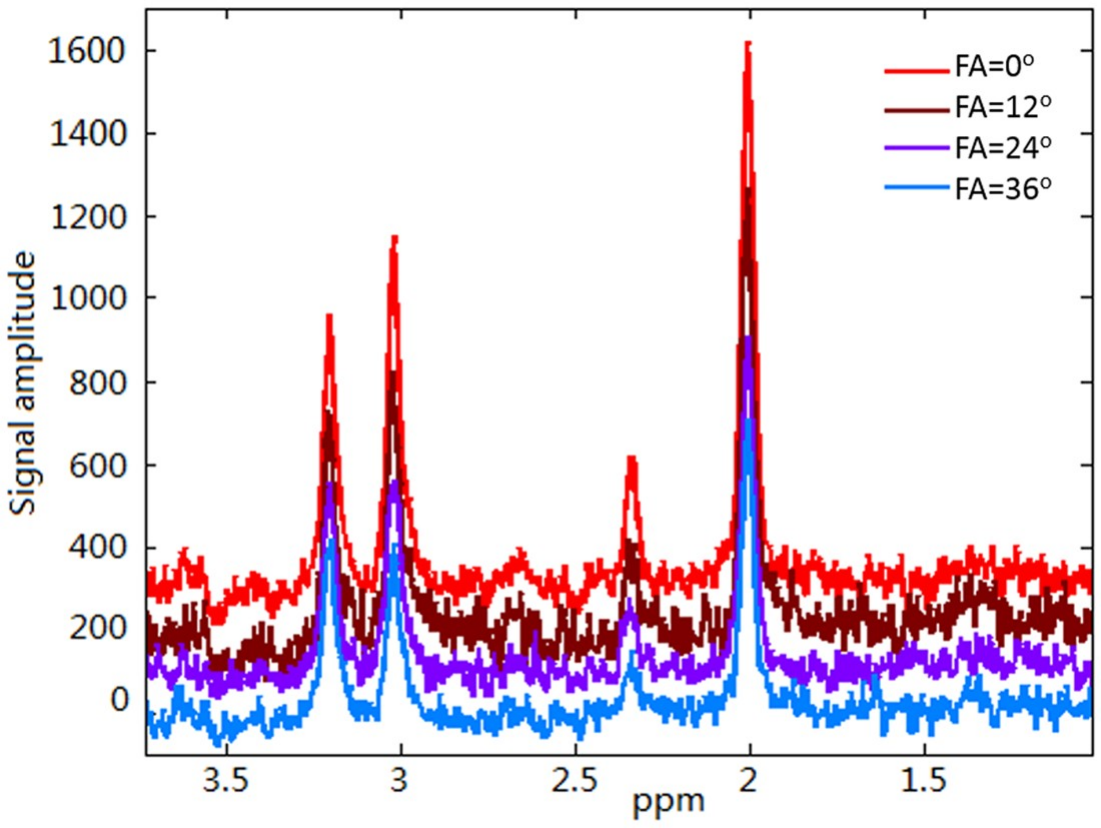
Typical raw in vivo spectra at different flip angles (FAs). Typical raw in vivo spectra acquired from the medial frontal lobe voxel of a healthy subject using the improved MARzss method at four different FAs.

**Fig 4:**
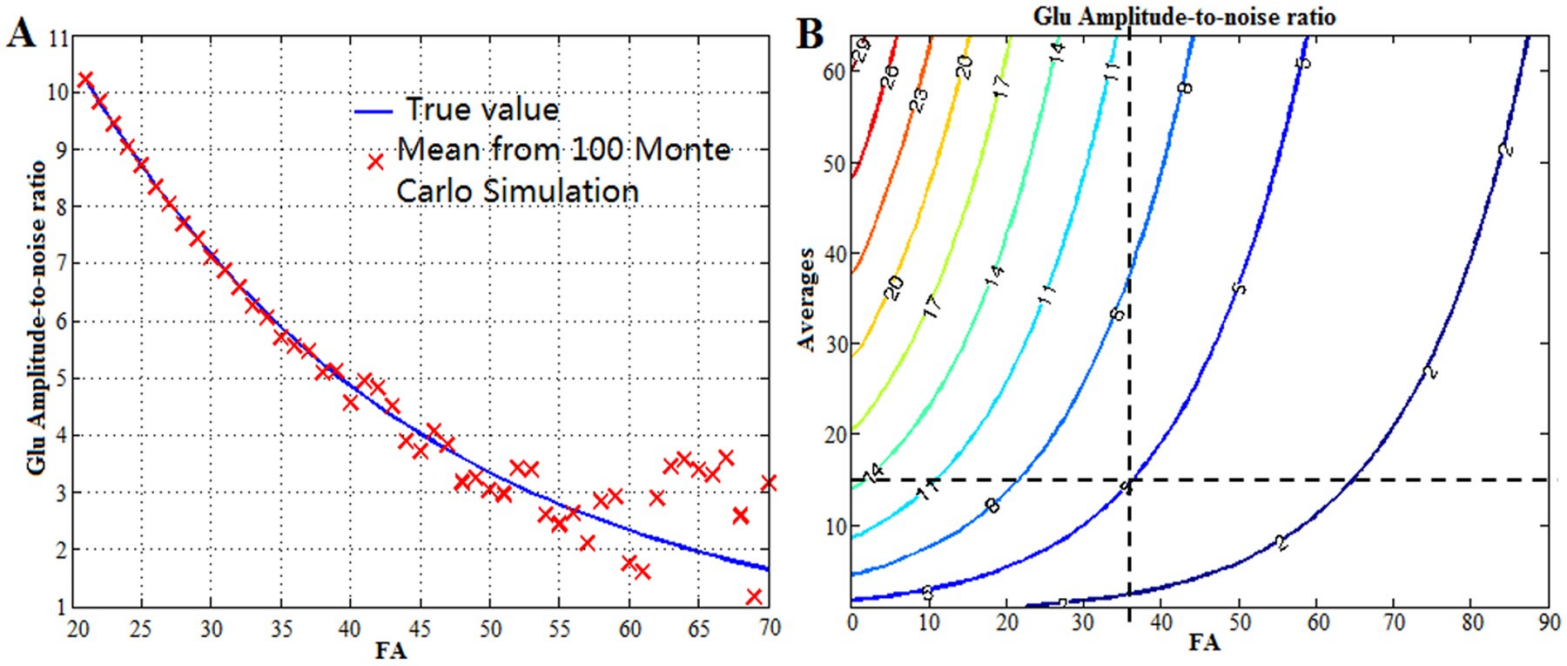
Monte Carlo simulation at different noise levels. (A) The mean value of glutamate (Glu) amplitude-to-noise ratio (red cross) from 100 Monte Carlo simulations at 50 different flip angles (FAs). Significant deviations between mean values from Monte Carlo simulations and ground truth values were observed when the Glu amplitude-to-noise ratio was below five. (B) The contour map of Glu amplitude-to-noise ratio as a function of number of signal averages and FA. Values of the ratio are labeled on each contour line. The dashed lines in D indicate that at FA = 36° the Glu amplitude to noise ratio was five when the number of averages = 16.

Fig 3 shows typical in vivo spectra without line-broadening acquired at four different FAs. Although noise intensities remained the same among the different FAs, SNR decreased significantly as the signal intensities decreased when FA increased. Fifty different SNR levels—corresponding to 50 different FAs varying between 20° to 70° degrees—were analyzed using Monte Carlo simulation. The mean and standard deviation of Glu amplitude-to-noise ratio from the 100 Monte Carlo simulations at each FA were calculated. Mean values were plotted using red x marks (see Fig 4A). The ground truth (blue line) was also plotted for comparison. Significantly more deviations in mean Glu amplitude-to-noise ratio from corresponding ground truth values were observed when the Glu amplitude-to-noise ratio was below five. As expected, standard deviations of Glu amplitude-to-noise ratio gradually increased when FA increased. A contour plot of Glu amplitude-to-noise as a function of the number of signal averages and FAs is shown in Fig 4B, which also provides guidance on how to choose the largest FA for measuring Glu T_2_. When 16 averages were used in the acquisition, the largest FA could be set to 36° (at the intersection of the two dashed lines in Fig 4B), so that the Glu amplitude-to-noise ratio was larger than five.

**Fig 5:**
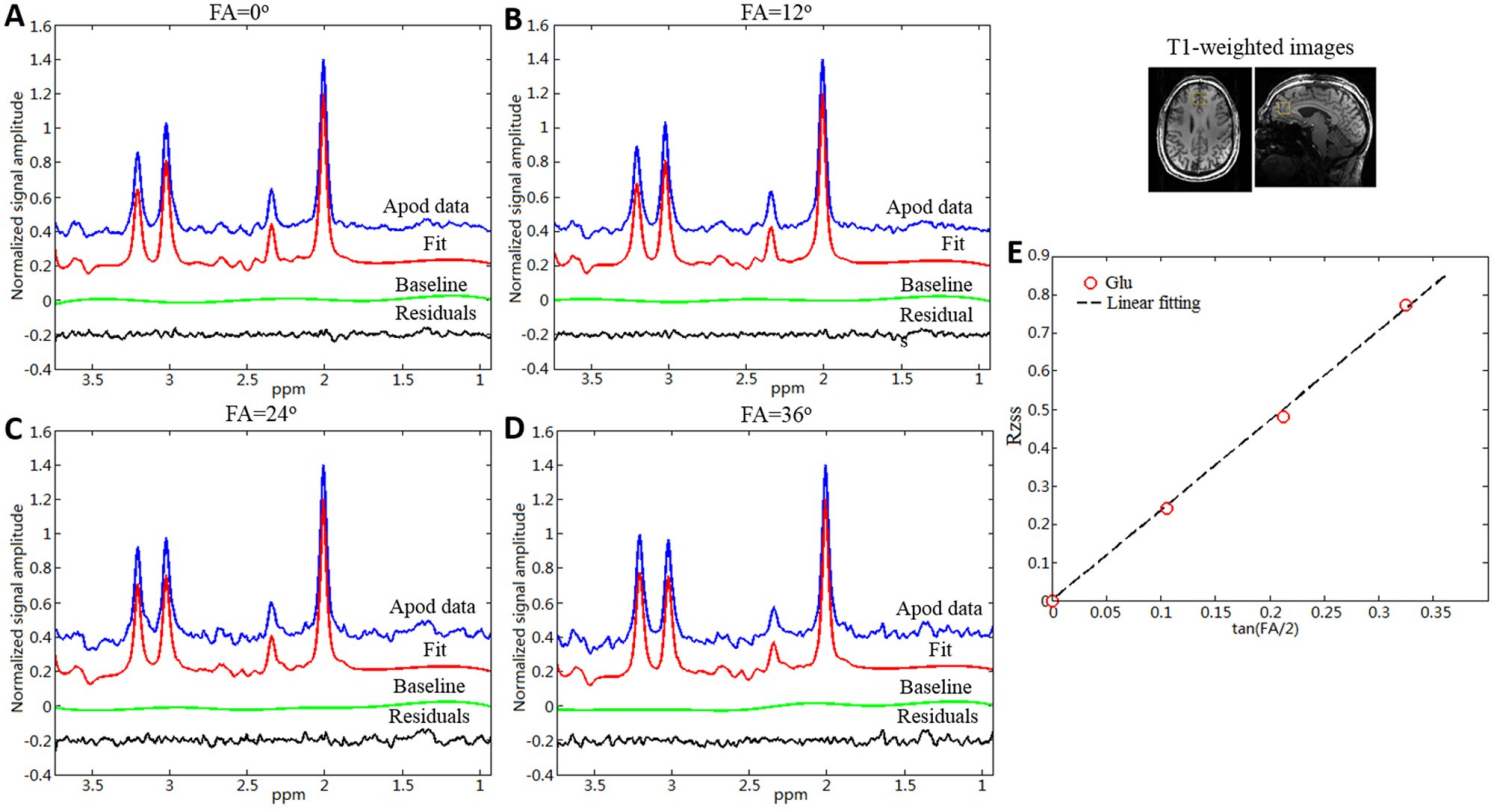
In vivo spectra fit and signal amplitude ratio fit. Individual fit of in vivo spectra acquired from the medial frontal lobe region of a healthy subject using the improved MARzss method at four different flip angles (FAs) (A,B,C, and D). All spectra were zero filled eight times and apodized using a combination of Lorentzian and Gaussian functions. Yellow squares were overlaid on the high resolution T_1_-weighted MRPAGE images at the right side of the spectra, indicating the position of the MRS voxel in a gray matter (GM)-dominated brain region. The signal amplitude ratio (Rzss) of glutamate (Glu) as a function of FA is plotted as a red circle in subplot E. Linear fitting is shown as a black dashed line.

The four FAs chosen for the in vivo study were 0, 12, 24, and 36° with 16 acquisition averages for each FA. TR_min_ of 8.5 secs, 7.5 secs, 6.0 secs, and 4.5 secs were used for FAs of 0°, 12°, 24° and 36°, respectively. By using a different TR_min_ for each FA, the total scan time for measuring Glu T_2_ was approximately seven minutes. Fig 5A–5D shows the linear combination fitting plots for individual spectra acquired from a voxel placed in the medial frontal lobe of a healthy subject at four different FAs. The voxel location is indicated by the yellow box overlaid on the axial and sagittal high resolution T_1_-weighted images. Threshold based-segmentation indicated that the averaged fraction of GM within the medial frontal lobe voxel was 65.0%. Note that the original data were zero-filled and apodized with Lorentzian and Gaussian functions. Glu intensities were used to calculate the in vivo signal amplitude ratio, *R*_*zss*_, defined in Eq [2].

Fig 5E displays *R*_*zss*_ as a function of tan(FA/2). With pre-determined Glu T_1_ values from the inversion recovery technique, Glu T_2_ was solved using Eqs [3] and [4]. The linear fitting coefficient of determination averaged over all subjects was 0.988. Similar spectral results were obtained from the left frontal and occipital lobes (see supporting information). Mean values and standard deviations for the T_1_ and T_2_ values of Glu in all three brain locations, as well as the averaged linear fitting coefficients of determination R^2^, are listed in Table 1. The mean T_2_ values of Glu from GM-dominated voxels were found to be longer than in WM-dominated voxels, consistent with previous studies [23,24]. Paired Student’s *t*-tests found that the T_2_ values of Glu from both the GM-dominated frontal lobe voxel and the GM-dominated occipital voxel region were significantly longer than the T_2_ values from the WM-dominated frontal lobe voxel (p = 0.0067 and p = 0.0040, respectively). In contrast, the mean T_1_ values of Glu from GM-dominated voxels were found to be shorter than those from WM-dominated voxels. Paired Student’s *t*-tests showed that the T_1_ values of Glu from the GM-dominated occipital lobe voxel were significantly shorter than those from the WM-dominated frontal lobe voxel (p = 0.021). No significant difference in T_1_ Glu values were found between GM-dominated frontal lobe voxel and WM-dominated frontal lobe voxel (p = 0.089). The standard deviations of T_2_ values of Glu were smaller in the frontal lobe region than in the occipital lobe region and were slightly greater than 10% across all three regions. The average peak linewidths were 13.8 Hz, 16.0 Hz, and 11.4 Hz in the GM-dominated medial frontal lobe region, the WM-dominated left frontal lobe region, and the GM-dominated medial occipital lobe region, respectively.

**Table 1:** In vivo glutamate *T*_*1*_ and *T*_*2*_ values.

	*T*_*1*_ (s)	*T*_*2*_ (ms)	*R*^*2*^	GM(%)	WM(%)	CSF(%)
**Medial frontal lobe**	1.15±0.14	117.5±12.9	0.988±0.012	65±4	22±3	13±2
**Left frontal lobe**	1.24±0.11	107.3±12.1	0.982±0.016	14±2	73±3	13±3
**Occipital lobe**	1.11±0.09	124.4±16.6	0.987±0.017	55±3	25±3	20±3

Glutamate *T*_*1*_ and *T*_*2*_ values measured from the frontal lobe gray matter (GM)-, frontal lobe white matter (WM)- and occipital lobe GM-dominated regions of healthy subjects*

* mean ± standard deviation. For both frontal lobe studies, n = 9. For the occipital lobe study, n = 8.

## Discussion

The present study made significant improvements to the previously developed MARzss method and applied it to characterize Glu relaxation in the frontal cortex. Bloch simulation suggested that both recovery delay time and RF pulse train duration could be shortened when the FA of the RF pulse train increased. However, when the FA is too large, the relatively weak signal intensity of Glu may become too small to be reliably determined. This study also determined experimental parameters for Glu T_2_ measurements based on Monte Carlo analysis and achievable SNR. The overall data acquisition time in this study was significantly shortened by using TR_min_ for each FA determined by Bloch simulation. Notably, the original MARzss method [12] used an iPFG train with fixed length and a long recycle delay and required more than 15 minutes to obtain enough data to conduct a T_2_ calculation if the number of averages was set to 16 for each FA. In contrast, the improved MARzss method used here significantly reduced the total scan time per voxel for the same SNR.

In this study, the in vivo transverse relaxation time of Glu was measured from three different brain locations using the improved MARzss method. Steady state longitudinal magnetization was prepared by iPFG train before the readout sequence in the MARzss method. Compared with the conventional multi-TE method, the MARzss method is largely free of undesirable TE-dependent variations, including scalar coupling modulations. There are few reported Glu T_2_ values measured from human subjects at 7 T (90–180 ms, see [23,24]) using the conventional multi-TE method. The Glu T_2_ values reported in the present study (95–141 ms) are within this 90–180 ms range, though closer to the lower end. It should be noted that with our improved MARzss method of determining Glu T_2_ at 7T, TE was fixed and the PRESS readout sequence was optimized for Glu detection with minimized spectral interference [19]. The acquisition time and FA were determined and optimized by Bloch and Monte Carlo simulations. Under these optimized experimental parameters, the standard deviation of Glu T_2_ was relatively small across all three brain regions. The T_2_ values obtained from the GM-dominated occipital lobe region were in general agreement with previously published values from the same region determined using the original MARzss technique [12], except that the standard deviation of Glu T_2_ in this study was significantly reduced. In addition, Monte Carlo simulation were performed to compare Glu T_2_ from the improved protocol with the original protocol. Under the same scan length, the improved protocol yielded an error or 1.2% while the original protocol yielded an error of 2.9%. Although noise in T_1_ measurements propagated into T_2_ measured by MARzss, the overall relative standard deviation of glutamate T_2_ after our optimization approximately matched those obtained using the conventional multi-TE method [23–25]. Furthermore, the requirements to measure T_1_ should not be considered a drawback because T_1_ relaxation per se may provide valuable information about cellular microenvironment of metabolites.

In our experiment we used a two-dimensional stimulated echo imaging experiment to calibrate the B_1_ value by placing the B_1_ null at the center of the prescribed voxel [20]. We did not quantify B_1_ inhomogeneity across the 2 x 2 x 2 cm^3^ voxel. However, B_1_ variation at 7 Tesla across a 2-cm length in human brain is very small. In addition, the effect of small variations in FA was linear in this study because our FA was much smaller than 90 degrees and thus still within the range of linear response theory. As a result, across the voxel the effect of B_1_ variations were largely canceled among themselves. Furthermore, given the increasing popularity of parallel transmission methods for B_1_ shimming at 7 Tesla, any residual errors due to B_1_ inhomogeneity can be further minimized [26].

Theoretically, the slope of a straight line is best determined by least squares fitting using two points, with the maximum separation between the two independent variables and the lowest noise level [13]. In the MRI literature, the relaxation times of tissue water have often been determined using only two points to shorten the total scan time. For example, in the state-of-the-art DESPOT1 and DESPOT2 methods (driven equilibrium single pulse observation of T_1_ and T_2_, respectively), linear expressions and two measurements per line were used to shorten scan time and reduce fitting errors [27]. In many in vivo situations, however, relaxation behavior may not be single exponential [28,29]. In these cases, more data points would be beneficial to reduce the bias associated with a single exponential model. In particular, relaxography techniques use a large number of data points to characterize water relaxation by multiexponential fitting [30]. Our spectroscopy voxels (8 ml) contained both GM and WM. The highly linear relationship observed experimentally between Rzss and tan (FA/2) (Fig 5) indicates that the two- or multi-exponential behavior of glutamate T_2_ relaxation partially due to tissue-type differences can be well-approximated by a phenomenological single exponential model within the range of our measurements. This observation is further corroborated by the fact that the Glu T_2_ values of the GM-dominated frontal and occipital voxels were not much longer than that of the WM-dominated frontal lobe voxel (Table 1). Nevertheless the statistically significant difference between those T_2_ values confirmed that Glu relaxation is truly multi-exponential even if one only considers the mixed tissue types in the voxel. Therefore it would be prudent to use more than two data points with equal statistical weighting for each point to fit the phenomenologically single exponential relationship (Eq [2]) to avoid unintended bias as the underlying multiexponential behavior is unknown. Further optimization of the acquisition parameter [31] based on the assumption of strictly single exponential relaxation, in our case, may create systematic bias as the underlying relaxation behavior for the much larger MRS voxel is not strictly single exponential.

Compared to the original MARzss method [12], the acquisition of MARzss data in this improved method was highly accelerated by using fewer FAs and a different TR_min_ for each FA, where TR_min_ for RF-driven steady state was pre-determined by Bloch simulation. The transverse relaxation information was computed based on linear regression of Eq [2]. As seen in Fig 2, the oscillatory behavior of the longitudinal magnetization quickly diminished after two seconds for the largest FA of 70°. For larger FAs, the Mz vs. iPFG duration curves stopped oscillating even earlier. This suggests that the duration of the iPFG train could be further reduced, and that Bloch or density matrix simulations could be used to predict a non-steady state Mz to extract relaxation information by nonlinear data fitting.

## Conclusion

In this work we significantly improved the MARzss method as evidenced by the shortened total scan times per voxel and significantly reduced standard deviations of Glu T_2_ values in vivo. In particular, we demonstrated that this improved MARzss method can be used in a typical clinical setting to reliably measure Glu T_2_ from multiple anatomical locations, including the technically more challenging frontal cortex.

## Supporting information

S1 Fig Density matrix simulated glutamate (Glu) spectra. Density matrix simulated Glu spectra with (blue) and without (red) interleaved RF and gradient pulses (iPFG) train at different flip angles (FAs). The top rows (A-C) are without line-broadening, and the bottom rows (D-F) are with line-broadening matching the in vivo linewidths. The readout sequence is a standard point-resolved spectroscopy (PRESS) sequence with amplitude-modulated excitation and refocusing radio frequency (RF) pulses and optimized echo time (TE) of [69, 37] ms. Differences between two Glu spectra are depicted in black at the bottom.
(TIF)

S2 Fig In vivo spectra from left frontal lobe. In vivo spectra acquired from the left frontal lobe region of a healthy subject using the improved MARzss method at four different flip angles (FAs). All spectra were zero-filled eight times and apodized using a combination of Lorentzian and Gaussian functions. Subplot A shows the stack view of four spectra. Yellow squares are overlaid on the high resolution T_1_-weighted MRPAGE images on top of the spectra, indicating the location of the MRS voxel in a white matter (WM)-dominated brain region. The individual fit of each spectrum is shown in subplots B, C, E, and F. The signal amplitude ratio (Rzss) of glutamate (Glu) as a function of FA is plotted as a red circle in subplot D. Linear fitting is shown as a black dashed line.
(TIF)

S3 Fig In vivo spectra from occipital lobe. In vivo spectra acquired from the occipital lobe region of a healthy subject using the improved MARzss method at four different flip angles (FAs). All spectra were zero-filled eight times and apodized using a combination of Lorentzian and Gaussian functions. Subplot A shows the stack view of four spectra. Yellow squares are overlaid on the high resolution T_1_-weighted MRPAGE images on top of the spectra, indicating the location of the MRS voxel location in a gray matter (GM)-dominated brain region. The individual fit of each spectrum is shown in subplots B, C, E, and F. The signal amplitude ratio (Rzss) of glutamate (Glu) as a function of FA is plotted as a red circle in subplot D. Linear fitting is shown as a black dashed line.
(TIF)

S1 File Density matrix simulation of iPFG pulse train. (DOCX)

S2 File In vivo results from left frontal lobe and occipital lobe. (DOCX)

S1 Table Mean values and standard deviations of Glu T_2_ from Monte Carlo simulations. Mean values and standard deviations of Glu T_2_ from Monte Carlo simulations with and without iPFG train. The ground truth Glu T_2_ was set to 98.0 ms. Abbreviations: Glu: glutamate; iPFG: interleaved RF and gradient pulses.
(DOCX)
