# Supplementary figures and images for: Quantification of in vivo transverse relaxation of glutamate in the frontal cortex of human brain by radio frequency pulse-driven longitudinal steady state
Source: PLoS One. 2019 Apr 17;14(4):e0215210. doi: 10.1371/journal.pone.0215210 (PMC6469797; doi:10.1371/journal.pone.0215210)

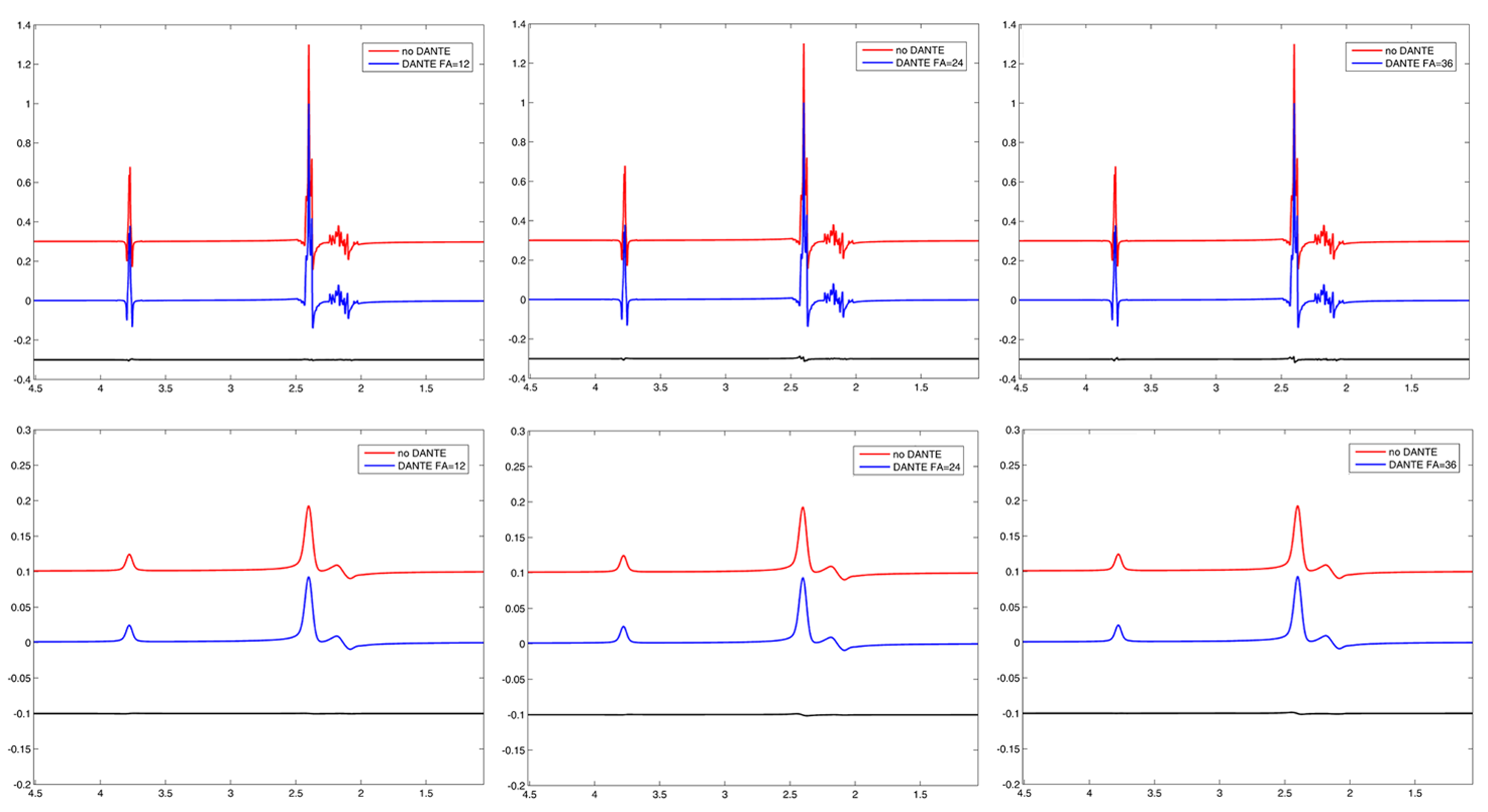

Supplement: S1 Fig — Density matrix simulated Glu spectra with (blue) and without (red) interleaved RF and gradient pulses (iPFG) train at different flip angles (FAs). The top rows (A-C) are without line-broadening, and the bottom rows (D-F) are with line-broadening matching the in vivo linewidths. The readout sequence is a standard point-resolved spectroscopy (PRESS) sequence with amplitude-modulated excitation and refocusing radio frequency (RF) pulses and optimized echo time (TE) of [69, 37] ms. Differences between two Glu spectra are depicted in black at the bottom. (TIF) [file pone.0215210.s001.tif]

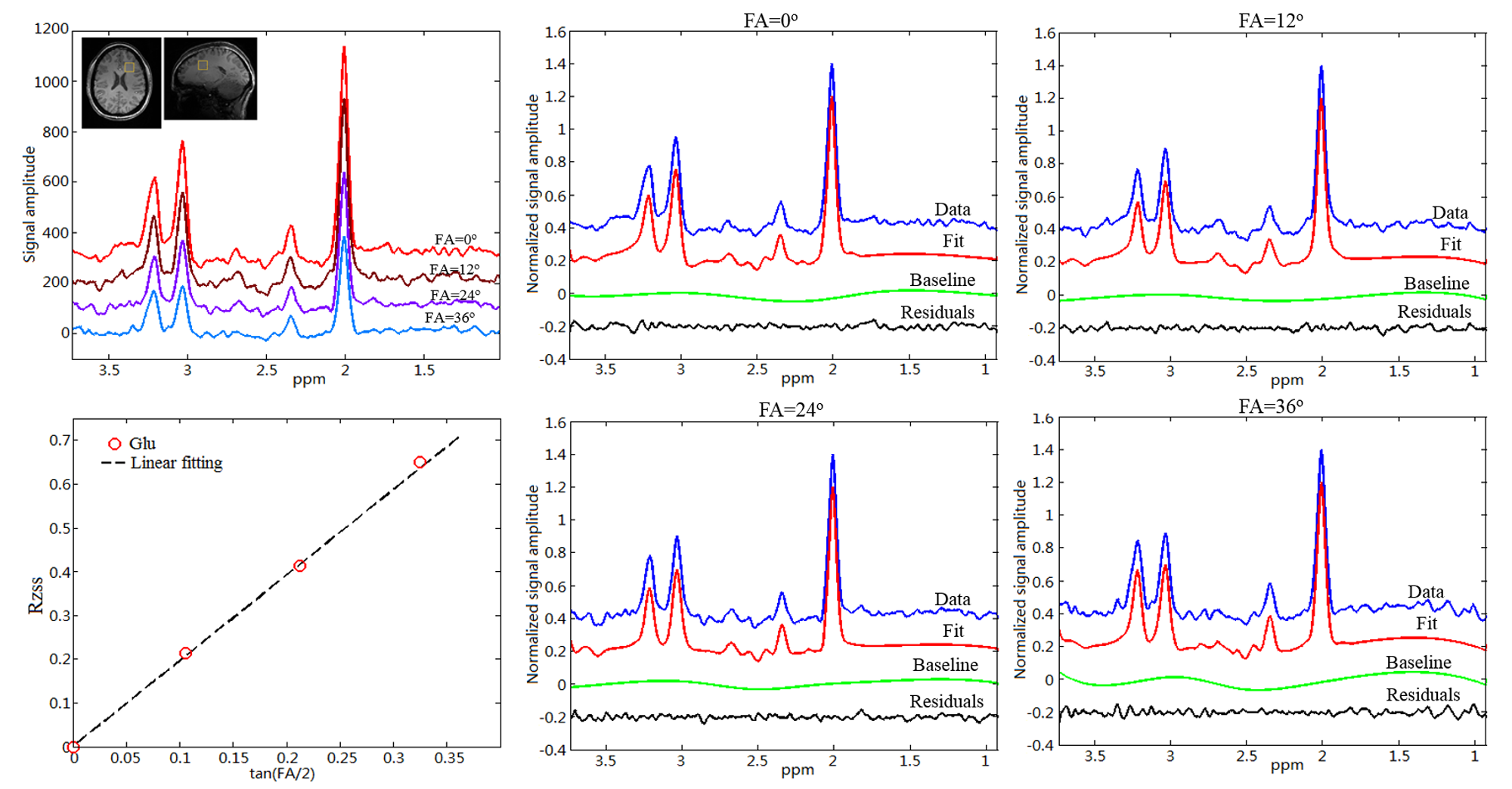

Supplement: S2 Fig — In vivo spectra acquired from the left frontal lobe region of a healthy subject using the improved MARzss method at four different flip angles (FAs). All spectra were zero-filled eight times and apodized using a combination of Lorentzian and Gaussian functions. Subplot A shows the stack view of four spectra. Yellow squares are overlaid on the high resolution T1-weighted MRPAGE images on top of the spectra, indicating the location of the MRS voxel in a white matter (WM)-dominated brain region. The individual fit of each spectrum is shown in subplots B, C, E, and F. The signal amplitude ratio (Rzss) of glutamate (Glu) as a function of FA is plotted as a red circle in subplot D. Linear fitting is shown as a black dashed line. (TIF) [file pone.0215210.s002.tif]

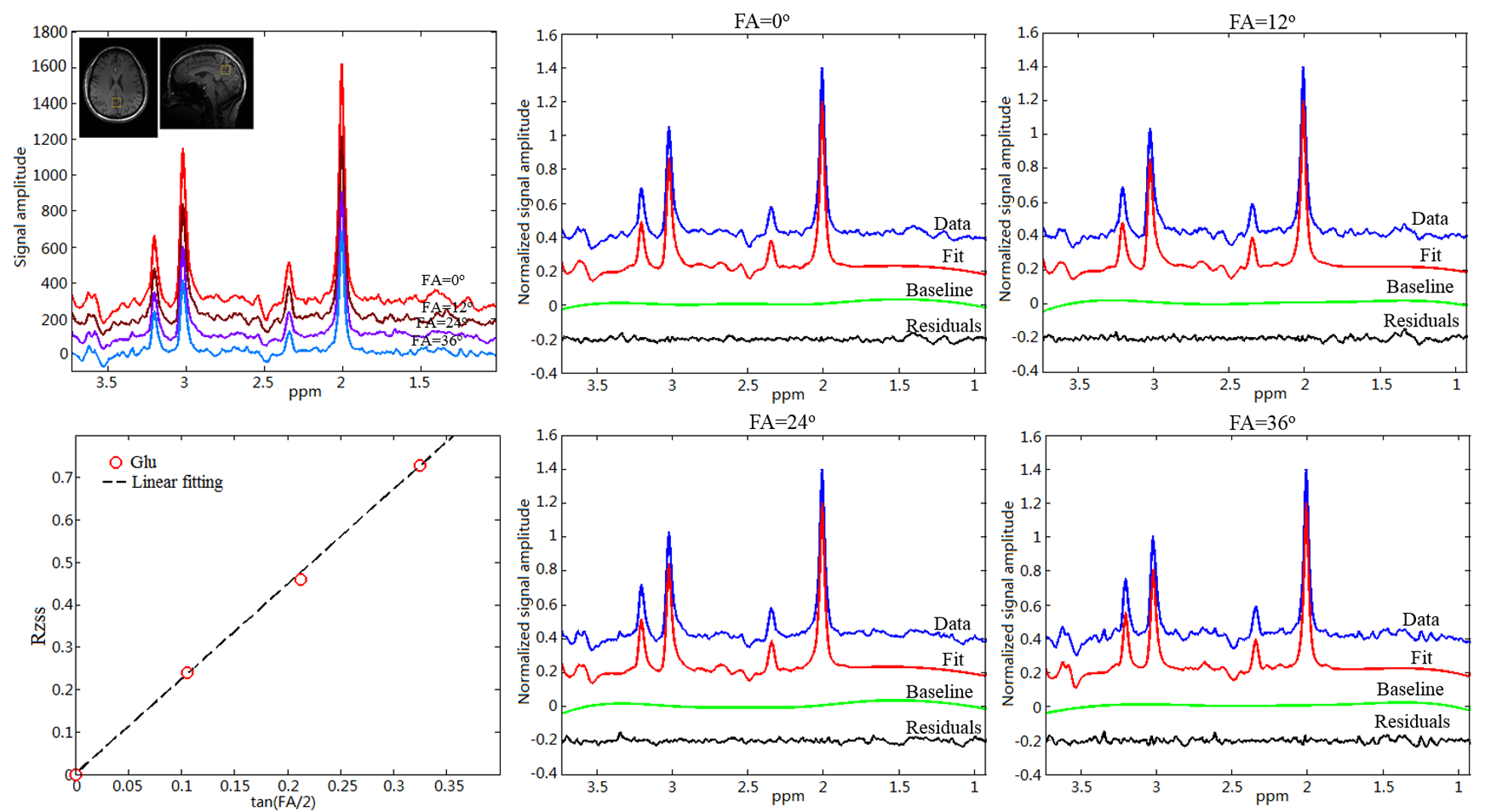

Supplement: S3 Fig — In vivo spectra acquired from the occipital lobe region of a healthy subject using the improved MARzss method at four different flip angles (FAs). All spectra were zero-filled eight times and apodized using a combination of Lorentzian and Gaussian functions. Subplot A shows the stack view of four spectra. Yellow squares are overlaid on the high resolution T1-weighted MRPAGE images on top of the spectra, indicating the location of the MRS voxel location in a gray matter (GM)-dominated brain region. The individual fit of each spectrum is shown in subplots B, C, E, and F. The signal amplitude ratio (Rzss) of glutamate (Glu) as a function of FA is plotted as a red circle in subplot D. Linear fitting is shown as a black dashed line. (TIF) [file pone.0215210.s003.tif]
