# Supplementary material for: Quantification of in vivo transverse relaxation of glutamate in the frontal cortex of human brain by radio frequency pulse-driven longitudinal steady state
Source: PLoS One. 2019 Apr 17;14(4):e0215210. doi: 10.1371/journal.pone.0215210 (PMC6469797; doi:10.1371/journal.pone.0215210)
Supplement: S1 File — (DOCX) [file pone.0215210.s004.docx]

**S1 File. Density matrix simulation of iPFG pulse train**

Phantom spectra of coupled spins including Glu and lactate when subjected to an iPFG train as implemented in MARzss showed no discernible changes in spectral pattern within the limits of experimental accuracy [12]. To quantify the effect of scalar evolution during the iPFG pulse train, numerical simulations of the Glu density matrix were performed to calculate the Glu spectrum. The iPFG train comprised 500 sinc-Gauss-shaped RF pulses with field gradients interspersed between the RF pulses. The readout sequence was a standard PRESS sequence with amplitude-modulated excitation and refocusing RF pulses [19]. TE was optimized as [69, 37] ms. The parameters of the simulated pulse sequence including RF and gradient pulses of the iPFG train, as well as delays between them, were identical to those used experimentally. A one-dimensional projection method was used to dramatically speed up the full density matrix simulation [20]. The results were compared with the Glu spectrum generated without the iPFG train. Monte Carlo simulations were used to evaluate the effect of scalar evolution on Glu quantification by fitting a synthetic dataset with known Glu concentrations using basis sets simulated with and without the iPFG train. The synthetic dataset used experimentally measured baseline and metabolite concentrations as described in the Methods.

The density matrix-simulated Glu spectra with and without the MARzss preparation module at three different FAs (12^o^, 24^o^, and 36^o^) is shown in S1 Fig A. The density matrix-simulated Glu spectra were also line-broadened (bottom figures) to match the in vivo linewidth. The difference between the simulated Glu spectra with and without the iPFG train are represented by the residual lines in black at the bottom. As shown in S1 Fig, the lineshapes of the simulated Glu spectra with and without the iPFG train were very similar. Monte Carlo simulation was performed to compare the Glu T_2_ obtained from fitting spectra using spectral basis calculated with and without the iPFG train, respectively. Noise level was determined from in vivo measurements with 16 averages. The mean values and standard deviations of Glu T_2_ from 100 Monte Carlo simulations with different noise realizations are listed in S1 Table. Two-tailed, unpaired Student’s *t*-tests showed no significant difference between Glu T_2_ obtained using the density matrix simulated with and without the iPFG train (p=0.16).
