# Supplementary material for: Quantification of in vivo transverse relaxation of glutamate in the frontal cortex of human brain by radio frequency pulse-driven longitudinal steady state
Source: PLoS One. 2019 Apr 17;14(4):e0215210. doi: 10.1371/journal.pone.0215210 (PMC6469797; doi:10.1371/journal.pone.0215210)
Supplement: S2 File — (DOCX) [file pone.0215210.s005.docx]

**S2 File. In vivo results from left frontal lobe and occipital lobe**

S2 Fig shows typical spectra obtained from a voxel placed in the left frontal lobe of a healthy subject at four different flip angles (FA). The voxel location is indicated by the yellow box overlaid on the axial and sagittal high resolution T_1_-weighted images on top of the spectra. A linear combination of fitting plots for individual spectra is displayed in S2 Fig B, C, E, and F. Glutamate (Glu) intensities were used to calculate the in vivo signal amplitude ratio, $R_{zss}$, defined in Eq. [2]. S2 Fig D displays $R_{zss}$ as a function of tan(FA/2). With pre-determined Glu T_1_ values from the inversion recovery technique, Glu T_2_ was solved using Eq. [3] and Eq. [4]. S3 Fig shows similar spectral results from the occipital lobe. One subject was excluded from the occipital lobe analysis because motion artifacts were observed. Threshold-based segmentation indicated that the average fraction of white matter (WM) in the voxel placed in the left frontal lobe was 73%, and the average fraction of the gray matter (GM) in the voxel placed in the occipital lobe was 55%.
