# Supplementary material for: Quantification of in vivo transverse relaxation of glutamate in the frontal cortex of human brain by radio frequency pulse-driven longitudinal steady state
Source: PLoS One. 2019 Apr 17;14(4):e0215210. doi: 10.1371/journal.pone.0215210 (PMC6469797; doi:10.1371/journal.pone.0215210)
Supplement: S1 Table — Mean values and standard deviations of Glu T2 from Monte Carlo simulations with and without iPFG train. The ground truth Glu T2 was set to 98.0 ms. Abbreviations: Glu: glutamate; iPFG: interleaved RF and gradient pulses. (DOCX) [file pone.0215210.s006.docx]

**S1 Table.** **Mean values and standard deviations of Glu T_2_ from Monte Carlo simulations.**

|  | **with iPFG train** | | **without iPFG train** | |
| --- | --- | --- | --- | --- |
|  | **Mean+SD** | **Err %** | **Mean+SD** | **Err %** |
| **Glu T2 (ms)** | 95.7+5.9 | 2.4 | 96.6+6.1 | 1.4 |

Mean values and standard deviations of Glu T_2_ from Monte Carlo simulations with and without iPFG train. The ground truth Glu T_2_ was set to 98.0 ms.

Abbreviations: Glu: glutamate; iPFG: interleaved RF and gradient pulses
